# Germacranolide-type sesquiterpene lactones from *Smallanthus sonchifolius* with promising activity against *Leishmania mexicana* and *Trypanosoma cruzi*

**DOI:** 10.1186/s13071-017-2509-6

**Published:** 2017-11-13

**Authors:** Jerónimo L. Ulloa, Renata Spina, Agustina Casasco, Patricia B. Petray, Virginia Martino, Miguel A. Sosa, Fernanda M. Frank, Liliana V. Muschietti

**Affiliations:** 1 Facultad de Farmacia y Bioquímica, Cátedra de Farmacognosia, IQUIMEFA (UBA-CONICET), Universidad de Buenos Aires, Junín 956 2° F (1113), Buenos Aires, Argentina; 2 Instituto de Histología y Embriología “Dr. Mario H. Burgos”, Facultad de Ciencias Médicas, Universidad Nacional de Cuyo-CONICET, (56 5500) Mendoza, CC Argentina; 3 CONICET, Instituto de Microbiología y Parasitología Médica (IMPaM), Universidad de Buenos Aires, Paraguay 2155 13° F (1211), Buenos Aires, Argentina; 4 Departamento de Microbiología, Facultad de Farmacia y Bioquímica, Inmunología y Biotecnología, Cátedra de Inmunología, Universidad de Buenos Aires, Junín 956 4° F (1113), Buenos Aires, Argentina

**Keywords:** Sesquiterpene lactones, *Smallanthus sonchifolius*, Leishmanicidal activity, Trypanocidal activity, In vitro assays, In vivo assays

## Abstract

**Background:**

Leishmaniasis and Chagas disease are life-threatening illnesses caused by the protozoan parasites *Leishmania* spp. and *Trypanosoma cruzi*, respectively. They are known as “neglected diseases” due to the lack of effective drug treatments and the scarcity of research work devoted to them. Therefore, the development of novel and effective drugs is an important and urgent need. Natural products are an important source of bioactive molecules for the development of new drugs. In this study, we evaluated the activity of enhydrin, uvedalin and polymatin B, three sesquiterpene lactones (STLs) isolated from *Smallanthus sonchifolius*, on *Leishmania mexicana* (MNYC/BZ/62/M) and *Trypanosoma cruzi* (Dm28c). In addition, the in vivo trypanocidal activity of enhydrin and uvedalin and the effects of these STLs on parasites’ ultrastructure were evaluated.

**Methods:**

The inhibitory effect of the three STLs on the growth of *L. mexicana* amastigotes and promastigotes as well as *T. cruzi* epimastigotes was evaluated in vitro. The changes produced by the STLs on the ultrastructure of parasites were examined by transmission electron microscopy (TEM). Enhydrin and uvedalin were also studied in a murine model of acute *T. cruzi* infection (RA strain). Serum activities of the hepatic enzymes alanine aminotransferase, aspartate aminotransferase and lactate dehydrogenase were used as biochemical markers of hepatotoxicity.

**Results:**

The three compounds exhibited leishmanicidal activity on both parasite forms with IC_50_ values of 0.42–0.54 μg/ml for promastigotes and 0.85–1.64 μg/ml for intracellular amastigotes. Similar results were observed on *T. cruzi* epimastigotes (IC_50_ 0.35–0.60 μg/ml). The TEM evaluation showed marked ultrastructural alterations, such as an intense vacuolization and mitochondrial swelling in both *L. mexicana* promastigotes and *T. cruzi* epimastigotes exposed to the STLs. In the in vivo study, enhydrin and uvedalin displayed a significant decrease in circulating parasites (50–71%) and no signs of hepatotoxicity were detected.

**Conclusions:**

Enhydrin, uvedalin and polymatin B possess significant leishmanicidal and trypanocidal activity on different parasite stages. These results show that these compounds may provide valuable leads for the development of new drugs against these neglected parasitic diseases.

## Background

Leishmaniasis is a poverty-associated, diverse and complex disease presenting several different forms, of which cutaneous leishmaniasis (CL) and visceral leishmaniasis (VL) are the most common. This disease is caused by different species of the kinetoplastid protozoan genus *Leishmania*, which is transmitted to humans by phlebotomine sand flies. Leishmaniasis occurs in 98 countries, where 300,000 new cases of VL and 1,000,000 new cases of CL are reported each year, with 350 million people living at risk [1]. The incidence of CL is increasing in Latin America and, despite it not being a life-threatening disease, it causes disability and leaves permanent scars that can lead to social prejudice [2]. Current treatments for CL are unsatisfactory; frequently, they are associated with significant failure rates and considerable toxicity. The World Health Organization recommends the use of pentavalent antimonials as first-line drugs [3]. However, these compounds are toxic and have poor effectiveness in the treatment of chronic manifestations of CL. Alternative treatments include the use of liposomal amphotericin-B (ANF); however, this drug cannot be widely employed due to its high cost and delivery requirements [4].

Chagas disease or American trypanosomiasis is a life-threatening illness caused by the parasite *Trypanosoma cruzi*. Annually, 56,000 new cases are reported in the Americas, and it is estimated that this disease causes about 12,000 deaths per year [5]. According to the Global Burden of Disease Study, Chagas disease was responsible for 550,000 disability adjusted life years in 2010 [6].

In many regions of Latin America, *T. cruzi* and *Leishmania* spp*.* infections overlap. Both species have close phylogenetic relationships [7] and therefore in these regions, it is sometimes difficult to differentiate serologically between Chagas disease and American CL due to antigenic cross-reactivity [8–10]. *Trypanosoma cruzi* strains are defined according to six different discrete typing units (DTUs TcI–TcVI) [11] which differ in virulence, pathogenicity, drug resistance, immune response, parasitaemia levels and tissue tropism [12–14].

Currently, there are two therapeutic options for the treatment of Chagas disease: the nitroheterocyclic compounds benznidazole (BNZ) and nifurtimox. These drugs were marketed in the 1970s but only BNZ has been recently approved by the Food and Drug Administration for use in children aged 2 to 12 years old with Chagas disease using the Accelerated Approval pathway [15]. BNZ is used as first line treatment because it has a better side-effect profile than nifurtimox. Nevertheless, both drugs have low or no efficacy during the chronic stage of the illness, have regional variations of effectiveness due to drug-induced resistance, and they present severe side effects that lead to the interruption of the treatment in a high percentage of patients [16]. Thus, there is an urgent need for the development of new drugs for the treatment of leishmaniasis and Chagas disease.

In the search of new therapeutic agents, natural products are an important source of bioactive molecules for the development of new drugs or as lead molecules which might enter into the drug discovery pipeline [17]. The importance of natural compounds, especially as antibacterial, antifungal, antiparasitic and antiviral agents, has been extensively reviewed by Newman & Cragg [18]. In the search for bioactive compounds with antiprotozoal activity [19–21], we have studied different plants, mainly from the family *Asteraceae*.

*Smallanthus sonchifolius* (*Asteraceae*) is a perennial species of South America commonly known as “yacón”. The tuberous roots of this plant have a sweet taste and are used as traditional food and eaten either raw alone or in fruit salads. They can be also boiled, baked or used to prepare beverages, syrup, juice or marmalade. The young stems are used as a vegetable similar to celery. The antidiabetic properties of *S. sonchifolius* are attributed to the leaves which are dried and used to prepare infusions [22, 23].

Literature data indicate that the leaves of this plant have antibacterial, anti-inflammatory and antifungal properties [24–26]. We have previously reported the cytotoxicity of six sesquiterpene lactones (STLs) isolated from *S. sonchifolius* in leukaemia and pancreatic cancer cells. Interestingly, one of those STLs was reported for the first time in nature [27], whilst the in vitro trypanocidal activity of the STLs enhydrin, uvedalin and polymatin B has been previously reported by our group [28]. In view of the results obtained, and considering the current drawbacks related to the treatment with trypanocidal drugs, this study was aimed at determining whether the STLs enhydrin and uvedalin are active in an animal model of the infection. In addition, the in vitro efficacy of the compounds against *Leishmania mexicana* and the susceptibility of a different strain of *T. cruzi* to the three STLs were investigated. Finally, the ultrastructure alterations induced by these STLs on *T. cruzi* and *L. mexicana* were also studied by TEM.

## Methods

### General experimental procedures

Infrared (IR) spectra were recorded in an Alpha FT-IR Spectrometer (Bruker, Ettlingen, Germany). Proton nuclear magnetic resonance (^1^H-NMR) and carbon NMR (^13^C-NMR) experiments were performed in a Bruker DRX 400 MHz spectrometer set at 400 MHz (^1^H) or 100 MHz (^13^C) and electron ionization-mass spectrometry (EI-MS) experiments were performed on a ThermoQ Exactive mass spectrometer (Thermo Scientific, West Palm Beach, FL, USA).

### Plant material

The aerial parts of *S. sonchifolius* (clone LIEY 97–2), were collected in the province of Tucumán, Argentina (Centro Universitario “Horco Molle”, University of Tucumán 26°47'S, 65°19'W, 547 m a.s.l.) in April 2013. All procedures were performed in agreement with institutional guidelines from IQUIMEFA (UBA-CONICET). A voucher specimen (LIL 607176) is deposited at the Herbarium of Instituto “Miguel Lillo”, Tucumán, Argentina.

### Extraction procedure, isolation and identification of the STLs

*Smallanthus sonchifolius* leaves were processed by soaking in dichloromethane at room temperature for 30 min as previously described [28]. This extract was subjected to a bioassay-guided fractionation by sequential silica gel (70–230 mesh; Macherey-Nagel, Düren, Germany) column chromatography using a gradient of *n-*hexane (2 × 250 ml), *n*-hexane-ethyl acetate (1:1) (2 × 250 ml), ethyl acetate (2 × 250 ml), ethyl acetate-methanol (1:1) (2 × 250 ml), and methanol (2 × 250 ml) as mobile phase. From this fractionation, three bioactive compounds were isolated and identified by comparison of the spectral data (IR, EI-MS, ^1^H-NMR and ^13^C-NMR) with previously published reports [27, 28] as the STLs enhydrin, polymatin B and uvedalin. Purity analysis was carried out by High Performance Liquid Chromatography (Waters® 600 chromatographer with a photodiode-array detector, Massachusetts, USA) on a RP-18 column. The purity of each compound was invariably > 95%.

### Parasites and media

*Leishmania mexicana* promastigotes (MNYC/BZ/62/M strain) were kindly provided by Berta Franke de Cazzulo (IIB-INTECH; CONICET-UNSAM). Parasites were grown in either Grace or Diamond medium supplemented with 10% foetal bovine serum (FBS). *Leishmania* promastigotes at the logarithmic phase were used in most assays, and at the stationary phase for macrophage infection. *Trypanosoma cruzi* epimastigotes (Dm28c strain) were grown and maintained in culture according to Sülsen et al. [19]. Bloodstream trypomastigotes of *T. cruzi* (RA strain) were obtained as previously described [20].

### Animals

Inbred male CF-1 and female C3H/HeN mice were nursed at the Department of Microbiology, School of Medicine (University of Buenos Aires). Animals were housed, kept and provided with water and a standard diet [20].

### Evaluation of antiprotozoal activity in axenic culture parasites

*Leishmania* promastigotes and *T. cruzi* epimastigotes growth inhibition was evaluated by direct counting in a Neubauer chamber. Stock solutions of test compounds were prepared in dimethyl sulfoxide (DMSO). The final concentration of DMSO did not exceed 1%. The assay was performed by duplicate, in the presence of 0, 1, 2.5, 5 or 10 μg/ml of each compound. Cell density was adjusted to 2.0–2.5 × 10^6^ parasites/ml and cells were cultured in the presence of each compound for 24 and 48 h. ANF (1 μg/ml, ICN) or BNZ (5 μg/ml, Elea) were used as positive controls for *Leishmania* and *T. cruzi* growth inhibition, respectively. A solution of 1% DMSO was used as negative control. The percentage of inhibition was calculated at 48 h as: 100 – [(Number of treated parasites)/(Number of untreated parasites)] × 100.

### Chemotherapeutic effects of enhydrin and uvedalin on the intracellular amastigote forms of *Leishmania*

Murine peritoneal macrophages were isolated from 4 to 6-week-old CF-1 mice (*n* = 3) by peritoneal lavage with 5 ml of ice-cold Roswell Park Memorial Institute medium (RPMI) 1640 (Gibco, Rockville, MD, USA) containing 100 IU/ml penicillin, 100 μg/ml streptomycin and 10% FBS (complete RPMI). Cells (1.5 × 10^5^/well) were seeded on glass coverslips inside 24- well plates (Corning Inc., Corning, NY, USA) and incubated overnight at 37 °C in 5% CO_2_. Non-adherent cells were washed away and the adherent macrophages were infected for 4 h with stationary-phase *Leishmania* promastigotes at a parasite/macrophage ratio of 5:1. After removal of extracellular parasites, cultures were incubated for 72 h in the presence of seven different concentrations of enhydrin or uvedalin ranging from 0 to 50 μg/ml. ANF was used as a positive control (0 to 0.6 μg/ml). The number of infected cells and the number of amastigotes per 100 cells were calculated by counting at least 200 cells per Giemsa-stained coverslip. Results were expressed as % anti-amastigote activity (% AA) = [1 – (Number amastigotes/100 treated cells) / (Number amastigotes/100 untreated cells)] × 100. IC_50_ values were determined for each compound. Cells were observed and photographed with a light microscope with an Olympus (Hachioji, Japan) microphotographic system at 400× magnification.

### In vivo trypanocidal activity of enhydrin and uvedalin

Groups of male C3H/HeN mice (6–10 mice/group, 6–8 week-old, 23–25 g weight), were inoculated with 5 × 10^3^ bloodstream *T. cruzi* trypomastigotes (RA strain) by the intraperitoneal (ip) route. After infection was established, on day 7 post-infection (pi), different groups of mice were treated with 1 mg/kg of body weight/day of either enhydrin, uvedalin or BNZ by the ip route for five consecutive days as previously reported [21]. Stock solutions of the compound samples were prepared in ethanol:water (1:1) and dilutions were made in PBS (pH 7.2). Another group of mice was treated with PBS (pH 7.2) as negative control. The final concentrations of ethanol did not exceed 0.1%. The parasitaemia was individually monitored every 2 days from day 5 to day 40 pi, in 5 μl of blood, by direct microscopic counting of parasites in a Neubauer chamber after the lysis of red cells. Body weight and clinical conditions, including physical appearance, were recorded every other day. Mortality was recorded every day until the end of the experiment (100 days) and the results were expressed as percentage of surviving animals [20].

### In vivo toxicity study

The in vivo toxicity of enhydrin and uvedalin was assessed in groups of 5 uninfected male C3H/HeN mice with a five-day course of daily ip injection of 1 mg/kg of body weight/day of the compounds. Stock solutions of the samples were prepared in ethanol:water (1:1) and dilutions were made in PBS (pH 7.2). PBS was also employed as negative control. After the fifth dose, blood samples were collected and the serum separated. Alanine aminotransferase (ALT), aspartate aminotransferase (AST) and lactate dehydrogenase (LDH) levels were used as biochemical markers to evaluate hepatotoxicity. Assays were carried out by ultraviolet spectrophotometry (Wiener Lab, Buenos Aires, Argentina).

### Transmission electron microscopy

*Leishmania* promastigotes and *T. cruzi* epimastigotes were treated with 2.5 and 0.5 μg/ml of enhydrin, polymatin B or uvedalin for 24 h. Ultrathin sections of parasite cells treated with these compounds were observed with a transmission electron microscope as previously described [20].

### Statistical analysis

Statistical analyses were carried out using GraphPad Prism 5 software (GraphPad, San Diego, CA, USA). In experiments with more than two groups of mice, data were analysed using one-way ANOVA with Dunnett’s post-test. If necessary, a logarithmic transformation was applied to obtain data with a normal distribution and 50% inhibitory concentration (IC_50_) values were estimated by linear regression as previously described [20]. A *P-*value lower than 0.05 was considered significant. Results were expressed as means ± SEM for each group. In survival experiments, curves were generated with GraphPad Prism 5 software and analysed with the log-rank test.

## Results

### In vitro activity against axenically cultured parasites

**Fig. 1:**
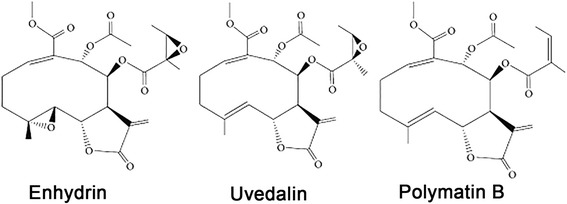
Chemical structures of enhydrin, uvedalin and polymatin B

**Fig. 2:**
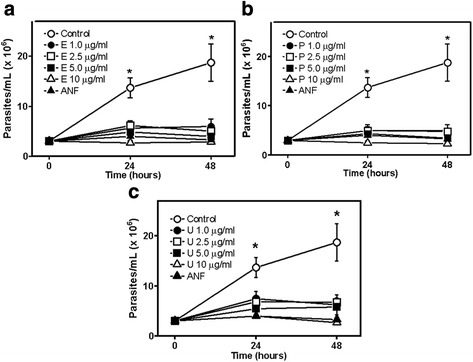
Effect of enhydrin (**a**), polymatin B (**b**) and uvedalin (**c**) on the growth of *L. mexicana* promastigotes at the indicated concentrations, after 24 h and 48 h of treatment. Symbols represent mean ± SEM from three independent experiments. ANF (1 μg/ml) was used as positive control and 1% DMSO was used as negative control. (*) above the means indicate significant differences between control *vs* all treatments at each time point (*P* ≤ 0.05)

**Fig. 3:**
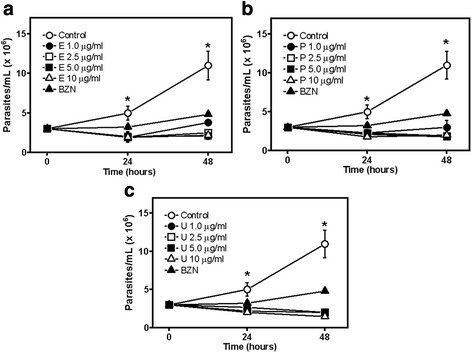
Effect of enhydrin (**a**), polymatin B (**b**) and uvedalin (**c**) on the growth of *T. cruzi* epimastigotes (Dm28c) at the indicated concentrations, after 24 and 48 h of treatment. Symbols represent mean ± SEM from three independent experiments. BZN (5 μg/ml) was used as positive control and 1% DMSO was used as negative control. (*) above the means indicate significant differences (*P* ≤ 0.05) between control and compounds under study at 24 (a) and 48 h (b)

The effect of the STLs enhydrin, polymatin B and uvedalin (Fig. 1) on the growth of *L. mexicana* promastigotes is shown in Fig. 2. The three compounds exhibited leishmanicidal activity with IC_50_ values of 0.43 μg/ml (0.92 ± 0.18 μM), 0.45 μg/ml (1.04 ± 0.25 μM) and 0.42 μg/ml (0.93 ± 0.2 μM) for enhydrin, polymatin B and uvedalin, respectively, with no significant differences among them. The inhibitory activity of the compounds on *T. cruzi* epimastigotes of DTU I (Dm28c strain, Fig. 3) was also evaluated. Enhydrin, polymatin B and uvedalin were active with IC_50_ values of 0.36 μg/ml (0.78 ± 0.22 μM), 0.60 μg/ml (1.38 ± 0.5 μM) and 0.35 μg/ml (0.79 ± 0.12 μM), respectively. All the compounds were more effective than BNZ (IC_50_ = 10.6 μM) and ANF (IC_50_ = 2 μM), used as positive control drugs for *T. cruzi* and *L. mexicana,* respectively.

### Growth inhibition of the intracellular stage of *Leishmania* spp.

**Fig. 4:**
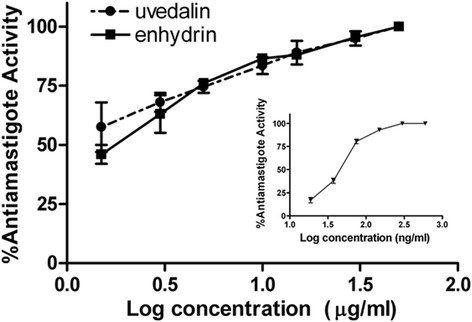
Inhibitory activity of uvedalin and enhydrin on *L. mexicana* amastigotes. Murine peritoneal macrophages were infected with stationary-phase *Leishmania* promastigotes (parasite:cell ratio 5:1). After washing out the unbound parasites, STLs were added at concentrations ranging from 0 to 50 μg/ml. Results were expressed as % antiamastigote activity (% AA). Inset: inhibitory activity of amphotericin B on *L. mexicana* intracelular amastigotes. Values represent the mean ± SEM. No significant differences were observed between both STLs

The chemotherapeutic effect of enhydrin and uvedalin was also studied on amastigotes. The inhibitory activity of the compounds is shown in Fig. 4. The IC_50_ values for enhydrin and uvedalin were 1.64 μg/ml (3.66 μM) and 0.85 μg/ml (1.89 μM), respectively. Amphotericin B presented an IC_50_ value of 3.35 ng/ml (3.62 nM) for amastigotes after 72 h of treatment.

### In vivo trypanocidal activity of enhydrin and uvedalin

**Fig. 5:**
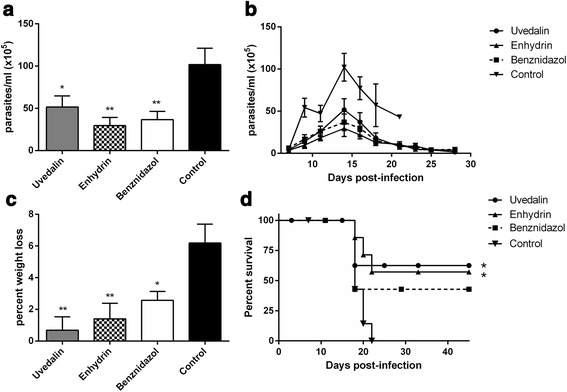
In vivo trypanocidal activity of uvedalin and enhydrin. Mice were infected with 5 × 10^3^ bloodstream trypomastigotes (RA strain) and treated for 5 consecutive days, starting on day 7 pi with 1 mg/kg/day of uvedalin, enhydrin, BNZ or with an equivalent volume of PBS (negative control). **a** Circulating parasites on the peak of parasitaemia (day 14 pi). **b** Parasitaemia curve. **c** Percentage of body weight loss on the peak of parasitaemia (on day 14 pi). **d** Survival curve during the acute infection period. Results are shown as mean ± SEM and are representative of 3 independent experiments (*P < 0.05, **P < 0.01 *vs* negative control)

Untreated mice inoculated with *T. cruzi* trypomastigotes (RA strain) displayed high parasitaemia levels with 100% mortality on day 22–25 post-infection (pi). When animals were treated with either enhydrin or uvedalin (for 5 consecutive days), a significant reduction of circulating parasites during the acute phase of *T. cruzi* infection was observed (Fig. 5a, b). Trypomastigotes were detected in all animals by 7 days pi, peaking on day 14 pi and declining gradually to low values by day 35 pi in the surviving animals. The mean peak parasitaemia for untreated mice was 1.0 ± 0.2 × 10^7^ parasites/ml. Uvedalin and enhydrin-treated mice presented a 50 and 71% reduction in the number of blood parasites, respectively (on day 14 pi), similar to the reduction observed in the BNZ-treated group (64%). No significant differences were observed between treatments (Mann-Whitney U-test uvedalin *vs* enhydrin, *U* = 8.5, *P* = 0.27; uvedalin *vs* BNZ, *U* = 14, *P* = 0.57; and enhydrin *vs* BNZ, *U* = 12, *P* = 0.65).

Those animals presenting higher number of circulating parasites also presented a higher body weight loss (Fig. 5c). Untreated animals presented a 6% body weight loss during the peak of parasitaemia, followed by death. Enhydrin and uvedalin-treated mice lost less than 1.5% of their body weight. The clinical condition of all animals improved during the treatment as well as that of animals treated with BNZ. By the end of the experiment (100 days pi), 57 and 52% of uvedalin and enhydrin-treated mice had survived (Fig. 5d). Regardless of the treatment, all surviving animals appeared physically healthy and displayed an active behaviour. All mice lost weight around the maximum parasitaemia time, especially non-treated animals, with no statistical differences between treated groups.

### In vivo toxicity of enhydrin and uvedalin

No signs of toxic effects were observed in uninfected mice during the 5 days of treatment with enhydrin and uvedalin. No significant body weight drop or death in any treatment group of uninfected mice was observed either. No significant differences were observed in AST, LDH and ALT serum activities between control and treatment groups.

### Transmission electron microscopy (TEM)

**Fig. 6:**
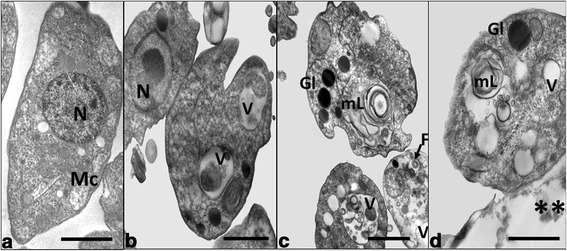
Ultrastructural effects of the STLs on *L. mexicana* promastigotes. Parasites were incubated with medium alone (**a**) as negative control or 0.5 μg/ml of either enhydrin (**b**), polymatin B (c) or uvedalin (**d**). Magnification: 3200× (**b**-**d**); 3600× (**a**). *Scale*-*bar*: 1 μm. *Abbreviations*: *N* nucleus, *Mc* mitochondria, *F* flagellum, *V* vacuoles, *GI* glycosomes, *mL* multilamellar structure; **, destroyed parasite

Ultrathin sections of *T. cruzi* epimastigotes and *Leishmania* promastigotes treated with enhydrin, polymatin B and uvedalin were analysed by TEM. Results demonstrated that *Leishmania* parasites were more sensitive to the compounds than *T. cruzi,* even at doses as low as 0.5 μg/ml. In *Leishmania*, the presence of multilamellar structures resembling cytoplasmic bodies and parasite lysis were observed (Fig. 6). At higher doses (2.5 μg/ml), both *L. mexicana* and *T. cruzi* suffered major ultrastructural alterations, such as intense vacuolization, with a higher proportion of parasites being destroyed (data not shown). In addition, mitochondrial swelling was observed on *T. cruzi* after the treatment with 2.5 μg/ml of uvedalin.

## Discussion

In earlier studies, we have reported the antiparasitic activity of different STLs isolated from Argentine medicinal species [19–21, 28]. There are over 5000 known structures of these naturally occurring compounds which are mainly present in members of the family *Asteraceae*. Their vast chemical diversity has been related to a wide range of biological activities ranging from antitumour, antibacterial, antihelminthic, uterus contracting, antimalarial and neurotoxic activity [29]. Artemisinin, a STL isolated from the leaves of the Chinese plant *Artemisia annua*, is considered the current gold-standard treatment for malaria, being the most relevant example of a STL with antiprotozoal activity [30].

In this study, we have demonstrated the leishmanicidal and trypanocidal activities of enhydrin, uvedalin and polymatin B, three STLs isolated from *S. sonchifolius* that belong to the germacranolide group. Results showed that the three compounds exhibited significant leishmanicidal activity against promastigotes of *L. mexicana* (IC_50_ values = 0.42–0.54 μg/ml). A vast number of natural products have been reported to show antileishmanial activity [31, 32]. Our results are comparable to other STLs, including those reported by us in previous studies [19, 20]. Considering that amastigotes are the clinically relevant stage of the parasite and that they are present in mammalian hosts, including humans, we have evaluated the activity of enhydrin and uvedalin on this intracellular stage (polymatin B was not included due its low content in the plant material). Both compounds inhibited amastigotes growth with IC_50_ values of 1.64 and 0.85 μg/ml, respectively. To date, no reports have described the antileishmanial activity of these compounds.

In a previous study, we have described the isolation of enhydrin, uvedalin and polymatin B by bioassay-guided fractionation from a crude extract of *S. sonchifolius* and the activity of these compounds on the RA strain of *T. cruzi* [28] that belongs to the DTU VI [11]. Since *T. cruzi* is genetically highly diverse, here we investigated the in vitro activity of these germacranolides on a strain belonging to DTU I (Dm28c), which is the most frequent of all *T. cruzi* DTUs in the Americas [12]. Although DTU VI is by far the most commonly used strain for in vitro assays [33], DTU I is the most common genotype associated with acute Chagas disease, and often found in chronic chagasic patients with cardiomyopathy [11]. Our results indicated that enhydrin, polymatin B and uvedalin are active against *T. cruzi* epimastigotes belonging to the Dm28c strain (DTU I). These compounds proved to be more active than BNZ (Fig. 3). The activity of the STLs on different strains of *T. cruzi* is important for the development of new antichagasic drugs since the variability in the morbidity of the disease is believed to be associated with the high complexity of parasite populations. Moreover, co-infections with *Leishmania* spp. and *T. cruzi* have been reported [8–10] and thus far, a single drug that is active against both parasites is not available.

Numerous plant metabolites have been shown to possess in vitro activity against *T. cruzi*; however, in vivo bioassays are scarce [34]. In this work, we have demonstrated that the administration of 1 mg/kg/day of enhydrin or uvedalin to *T. cruzi*-infected mice produce a significant reduction in parasitaemia, similar to the reduction observed in BNZ-treated animals. The treatment with enhydrin or uvedalin prevented the weight loss observed in PBS-treated mice. Although STLs seemed to be more effective than BNZ in terms of body weight loss prevention, no statistically significant differences were observed between them. It should be noted that although animals were treated for only five days with doses 2 log lower than those used in the conventional treatment with BNZ in the murine model, a significant decrease of mortality was observed in treated mice, as compared to the control group.

TEM ultrastructural analyses were carried out to assess the effect of the three STLs on *L. mexicana* and *T. cruzi*. The TEM evaluation demonstrated the existence of marked ultrastructural alterations in both parasites. The multilamellar structures, observed in *L. mexicana* promastigotes treated with 0.5 μg/ml of each compound could correspond to autophagic vacuoles that are probably part of an underlying mechanism of parasite death. On the other hand, after a 2.5 μg/ml treatment with enhydrin and polymatin B, an intense cytoplasmic vacuolization in both parasite species was observed. Uvedalin caused mitochondrial swelling in *T. cruzi* epimastigotes (data not shown). This finding is in line with those obtained with other STLs like psilostachyin [35] and parthenolide [36]. The fact that kinetoplastids have a single branched mitochondrion makes this organelle an attractive drug target [37]. Even though the mechanism of action of these STLs has not yet been determined, these compounds could exert their antiprotozoal effect through the generation of reactive oxygen species (ROS) [38, 39]. Further studies are needed to confirm this hypothesis.

In this study, drug-induced liver injury was assessed through the measurement of biomarkers. When uninfected animals were treated with the compounds, no signs of toxicity were observed. Moreover, considering the CC_50_ values previously reported by our group [28], the selectivity index values (SI) for *Leishmania* amastigotes were 12.7 and 24.8 for enhydrin and uvedalin, respectively. These data indicate that these compounds show good selectivity when tested against mammalian cells.

BNZ and nifurtimox can have different adverse effects, with adults being more susceptible than children. Furthermore, some patients are prone to treatment discontinuation due to the appearance of such severe adverse events [40]. These issues are relevant, since an intense debate still exists regarding the parameters for treatment success in Chagas disease.

## Conclusion

In conclusion, the leishmanicidal activity of enhydrin, uvedalin and polymatin B, on different forms of the parasites has been demonstrated for the first time. Further to this, we have demonstrated the trypanocidal activity of these compounds on a DTU I strain of *T. cruzi* as well as its in vivo trypanocidal potential. TEM studies revealed important ultrastructural alterations in *T. cruzi* and *Leishmania* parasites treated with the compounds. The dual leishmanicidal and trypanocidal activities shown by enhydrin and uvedalin could be an interesting advantage. Thus, these STLs could be considered as promising lead compounds for the development of new therapies for the treatment of Chagas disease and leishmaniasis.

## Abbreviations

^13^C-NMR: Carbon-13 nuclear magnetic resonance
^1^H-NMR: Proton nuclear magnetic resonance
ALT: Alanine aminotransferase
ANF: Amphotericin
AST: Aspartate aminotransferase
BNZ: Benznidazole
CL: Cutaneous leishmaniasis
DMSO: Dimethyl sulfoxide
EI-MS: Electron ionization-mass spectrometry
FBS: Foetal bovine serum
IR: Infrared
LDH: Lactate dehydrogenase
PBS: Phosphate buffered saline
Pi: Post-infection
RPMI: Roswell Park Memorial Institute
STLs: Sesquiterpene lactones
TEM: Transmission electron microscopy
VL: Visceral leishmaniasis

## References

[1] Alvar J, Vélez ID, Bern C, Herrero M, Desjeux Cano J, Jannin J, den Boe M (2012). WHO Leishmaniasis Control Team Leishmaniasis worldwide and global estimates of its incidence. PLoS One.

[2] Bern C, Maguire JH, Alvar J (2008). Complexities of assessing the disease burden attributable to leishmaniasis. PLoS Negl Trop Dis.

[3] World Health Organization (WHO) (2010). Control of the Leishmaniases. Report of a meeting of the WHO Expert Committee on the Control of Leishmaniases. WHO Tech Rep Ser.

[4] DNDi - Drugs for Neglected Diseases initiative. http://www.dndi.org/diseases-projects/leishmaniasis/. Accessed 10 May 2017.

[5] PAHO Pan-American Health Organization- http://www.paho.org/hq/index.php?option=com_topics&view=article&id=10&Itemid=4 0743. Accessed 10 May 2017.

[6] Murray CJ, Vos T, Lozano R, Naghavi M, Flaxman AD, Michaud C (2012). Disability-adjusted life years (DALYs) for 291 diseases and injuries in 21 regions, 1990–2010: a systematic analysis for the global burden of disease study 2010. Lancet.

[7] El-Sayed NM, Myler PJ, Blandin G, Berriman M, Crabtree J, Aggarwal G (2005). Comparative genomics of trypanosomatid parasitic protozoa. Science.

[8] Frank FM, Fernández MM, Taranto NJ, Cajal SP, Margni RA, Castro E (2003). Characterization of human infection by *Leishmania* spp. in the northwest of Argentina: immune response, double infection with *Trypanosoma cruzi* and species of *Leishmania* involved. Parasitology.

[9] Umezawa ES, Souza AI, Pinedo-Cancino V, Marcondes M, Marcili A, Camargo LM (2009). TESA-blot for the diagnosis of Chagas disease in dogs from co-endemic regions for *Trypanosoma cruzi*, *Trypanosoma evansi* and *Leishmania chagasi*. Acta Trop.

[10] Parodi C, García Bustos MF, Barrio A, Ramos F, González Prieto AG, Mora MC (2016). American tegumentary leishmaniasis: T-cell differentiation profile of cutaneous and mucosal forms-co-infection with *Trypanosoma cruzi*. Med Microbiol Immunol.

[11] Zingales B, Andrade SG, Briones MR, Campbell DA, Chiari E, Fernandes O (2009). A new consensus for *Trypanosoma cruzi* intraspecific nomenclature: second revision meeting recommends TcI to TcVI. Mem Inst Oswaldo Cruz.

[12] Zingales B, Miles MA, Campbell DA, Tibayrenc M, Macedo AM, Teixeira MM (2012). The revised *Trypanosoma cruzi* subspecific nomenclature: rationale, epidemiological relevance and research applications. Infect Genet Evol.

[13] Gruendling AP, Massago M, Teston APM, Monteiro WM, Kaneshima EN, Araújo SM (2015). Impact of benznidazole on infection course in mice experimentally infected with *Trypanosoma cruzi* I, II and IV. Am J Trop Med Hyg.

[14] Margioto Teston AP, Paula de Abreu A, Gruendling AP, Bahia MT, Gomes ML, Marques de Araújo S (2016). Differential parasitological, molecular, and serological detection of *Trypanosoma cruzi* I, II, and IV in blood of experimentally infected mice. Exp Parasitol.

[15] FDA - Food and Drug Administration approves first U.S. treatment for Chagas disease. https://www.fda.gov/NewsEvents/Newsroom/PressAnnouncements/ucm573942.htm. Accessed 4 September 2017.

[16] Bern C, Montgomery SP, Herwaldt BL, Rassi A, Marin-Neto JA, Dantas RO (2007). Evaluation and treatment of Chagas disease in the United States: a systematic review. JAMA.

[17] Jones AJ, Grkovic T, Sykes ML, Avery VM (2013). Trypanocidal activity of marine natural products. Mar Drugs.

[18] Newman DJ, Cragg GM (2016). Natural products as sources of new drugs from 1981 to 2014. J Nat Prod.

[19] Sülsen VP, Frank FM, Cazorla SI, Anesini CA, Malchiodi EL, Freixa B (2008). Trypanocidal and leishmanicidal activities of sesquiterpene lactones from *Ambrosia tenuifolia* Sprengel (Asteraceae). Antimicrob Agents Chemother.

[20] Sülsen VP, Frank FM, Cazorla SI, Barrera P, Freixa B, Vila R (2011). Psilostachyin C: a natural compound with trypanocidal activity. Int J Antimicrob Agents.

[21] Sülsen VP, Cazorla SI, Frank FM, Laurella LC, Muschietti LV, Catalán CA (2013). Natural terpenoids from *Ambrosia* species are active *in vitro* and *in vivo* against human pathogenic trypanosomatids. PLoS Negl Trop Dis.

[22] Ojansivu I, Ferreira CL, Salminen S (2011). Yacon, a new source of prebiotic oligosaccharides with a history of safe use. Trends Food Sci Technol.

[23] Genta SB, Cabrera WM, Mercado MI, Grau A, Catalán CA, Sánchez SS (2010). Hypoglycemic activity of leaf organic extracts from *Smallanthus sonchifolius*: constituents of the most active fractions. Chem Biol Interact.

[24] Inoue A, Tamogami S, Kato H, Nakazato Y, Akiyama M, Kodama O (1995). Antifungal melampolides from leaf extracts of *Smallanthus sonchifolius*. Phytochemistry.

[25] Lin F, Hasegawa M, Kodama O (2003). Purification and identification of antimicrobial sesquiterpene lactones from yacon (*Smallanthus sonchifolius*) leaves. Biosci Biotechnol Biochem.

[26] Schorr K, Merfort I, Da Costa FBA (2007). Novel dimeric melampolide and further terpenoids from *Smallanthus sonchifolius* (Asteraceae) and the inhibition of the transcription factor NF- κB. Nat Prod Commun.

[27] De Ford C, Ulloa JL, Catalán CA, Grau A, Martino VS, Muschietti LV, Merfort I (2015). The sesquiterpene lactone polymatin B from *Smallanthus sonchifolius* induces different cell death mechanisms in three cancer cell lines. Phytochemistry.

[28] Frank FM, Ulloa J, Cazorla SI, Maravilla G, Malchiodi EL, Grau A (2013). Trypanocidal activity of *Smallanthus sonchifolius*: identification of active sesquiterpene lactones by bioassay-guided fractionation. Evid Based Complement Alternat Med.

[29] Merfort I (2011). Perspectives on sesquiterpene lactones in inflammation and cancer. Curr Drug Targets.

[30] Fernández-Álvaro E, Hong WD, Nixon GL, O'Neill PM, Calderón F (2016). Antimalarial chemotherapy: natural product inspired development of preclinical and clinical candidates with diverse mechanisms of action. J Med Chem.

[31] Schmidt TJ, Khalid SA, Romanha AJ, Alves TM, Biavatti MW, Brun R (2012). The potential of secondary metabolites from plants as drugs or leads against protozoan neglected diseases - part I. Curr Med Chem.

[32] Schmidt TJ, Khalid SA, Romanha AJ, Alves TM, Biavatti MW, Brun R (2012). The potential of secondary metabolites from plants as drugs or leads against protozoan neglected diseases - part II. Curr Med Chem.

[33] Duran-Rehbein GA, Vargas-Zambrano JC, Cuéllar A, Puerta CJ, Gonzalez JM (2014). Mammalian cellular culture models of *Trypanosoma cruzi* infection: a review of the published literature. Parasite.

[34] Muschietti L, Sülsen VP, Martino VS, Rahman AU (2013). Bioprospection of potential trypanocidal drugs: a scientific literature survey over the period 2000–2010. Studies in natural products chemistry.

[35] Sülsen V, Barrera P, Muschietti L, Martino V, Sosa M (2010). Antiproliferative effect and ultrastructural alterations induced by psilostachyin on *Trypanosoma cruzi*. Molecules.

[36] Izumi E, Morello LG, Ueda-Nakamura T, Yamada-Ogatta SF, Dias Filho BP, Garcia Cortez DA (2007). *Trypanosoma cruzi*: antiprotozoal activity of parthenolide obtained from *Tanacetum parthenium* (L.) Schultz Bip. (Asteraceae, Compositae) against epimastigote and amastigote forms. Exp Parasitol.

[37] de Souza W, Attias M, Rodrigues JC (2009). Particularities of mitochondrial structure in parasitic protists (Apicomplexa and Kinetoplastida). Int J Biochem Cell Biol.

[38] Sülsen VP, Puente V, Papademetrio D, Batlle A, Martino VS, Frank FM, Lombardo ME (2016). Mode of action of the sesquiterpene lactones psilostachyin and psilostachyin C on *Trypanosoma cruzi*. PLoS One.

[39] Barrera P, Sülsen VP, Lozano E, Rivera M, Beer MF, Tonn C (2013). Natural sesquiterpene lactones induce oxidative stress in *Leishmania mexicana*. Evid Based Complement Alternat Med.

[40] Antinori S, Grande R, Bianco R, Traversi L, Cogliati C, Torzillo D (2015). High frequency of adverse reactions and discontinuation with benznidazole treatment for chronic Chagas disease in Milan, Italy. Clin Infect Dis.

